# Surgery in Acute Metastatic Spinal Cord Compression: Timing and Functional Outcome

**DOI:** 10.3390/cancers14092249

**Published:** 2022-04-30

**Authors:** Hanno S. Meyer, Arthur Wagner, Alessandra Raufer, Ann-Kathrin Joerger, Jens Gempt, Bernhard Meyer

**Affiliations:** Department of Neurosurgery, School of Medicine, Technical University of Munich, 81675 Munich, Germany; arthur.wagner@tum.de (A.W.); alessandra.raufer@t-online.de (A.R.); annkathrin.joerger@tum.de (A.-K.J.); jens.gempt@tum.de (J.G.); bernhard.meyer@tum.de (B.M.)

**Keywords:** functional outcome, neurological function, recovery, spinal metastasis, timing

## Abstract

**Simple Summary:**

Spinal metastases affect an exceptionally high number of cancer patients and thereby represent a common challenge for healthcare providers. Patients may suffer from debilitating symptoms, including excruciating back pain, immobility and even neurological dysfunction. An exceptionally acute clinical presentation is caused by the compression of the spinal cord through growth of a spinal metastasis within the spinal canal, which may leave the patient with acute spinal cord injury in need of rapid surgical treatment. In clinical practice and science, no true timeframe has yet been defined within which these patients need to undergo surgery, although it is generally understood that their recovery and functional rehabilitation correlate with the time to surgery after symptom onset. In our study, we analyzed a surgically treated cohort of patients with acute spinal cord injury by metastatic compression to investigate the correlation of the timing of surgery with neurological recovery. We were able to identify a subgroup of patients with significantly improved recovery, in whom surgery was initiated within 16 h after admission. Complication rates were not significantly more frequent in this subgroup compared to patients operated on after 16 h. Based on these findings, we conclude that striving for surgery as early as feasible is a warranted strategy in patients with acute neurological deterioration due to metastatic spinal cord compression.

**Abstract:**

*Background:* Patients with metastatic spinal cord compression (MSCC) may experience long-term functional impairment. It has been established that surgical decompression improves neurological outcomes, but the effect of early surgery remains uncertain. Our objective was to evaluate the impact of early versus late surgery for acute MSCC due to spinal metastases (SM). *Methods:* We retrospectively reviewed a consecutive cohort of all patients undergoing surgery for SMs at our institution. We determined the prevalence of acute MSCC; the time between acute neurological deterioration as well as between admission and surgery (standard procedure: decompression and instrumentation); and neurological impairment graded by the ASIA scale upon presentation and discharge. *Results:* We screened 693 patients with surgery for spinal metastasis; 140 patients (21.7%) had acute MSCC, defined as neurological impairment corresponding to ASIA grade D or lower, acquired within 72 h before admission. Non-MSCC patients had surgery for SM-related cauda equina syndrome, radiculopathy and/or spinal instability. Most common locations of the SM in acute MSCC were the thoracic (77.9%) and cervical (10.7%) spine. Per standard of care, acute MSCC patients underwent surgery including decompression and instrumentation, and the median time from admission to surgery was 16 h (interquartile range 10–22 h). Within the group of patients with acute MSCC, those who underwent early surgery (i.e., before the median 16 h) had a significantly higher rate of ASIA improvement by at least one grade at discharge (26.5%) compared to those who had late surgery after 16 h (10.1%; *p* = 0.024). Except for a significantly higher sepsis rate in the late surgery group, complication rates did not differ between the late and early surgery subgroups. *Conclusions:* We report data on the largest cohort of patients with MSCC to date. Early surgery is pivotal in acute MSCC, substantially increasing the chance for neurological improvement without increasing complication rates. We found no significant impact when surgery was performed later than 24 h after admission. These findings will provide the framework for a much-needed prospective study. Until then, the treatment strategy should entail the earliest possible surgical intervention.

## 1. Introduction 

Spinal metastases are the most commonly encountered spinal tumors and have gained considerable epidemiological significance in recent decades. Advances in standards of care and targeted systemic therapies constituted a substantial increase in life expectancy, which in turn led to an increasing incidence of MSCC that has been reported to occur in 5–14% of all cancer patients [1,2]. Modern interdisciplinary treatment paradigms take cancer biology, the prospective survival, and the functional status of the patient into account [2,3]. Patients with MSCC frequently suffer not only from pain, but also from neurological deficits and impaired functional autonomy [4,5]. This negatively impacts their ability to undergo adjunct treatment and thus overall survival [6,7,8,9]. It has long been established that patients with symptomatic MSCC benefit from surgical decompression in addition to radiotherapy with regards to their functional outcome and possibly also to their life expectancy [4,9]. Based on the underlying pathophysiological concepts of spinal cord damage by a growing epidural mass with rapid neurological deterioration, that is, direct pressure and ischemia as well as tumor-related spinal instability, it is obvious that timing is critical, similar to the timing of surgery in spinal trauma. This has prompted most neurosurgical services to adopt a strategy aiming at timely surgery [3,10,11]. However, there are only few studies comparing early versus late surgery in acute symptomatic MSCC to date, and the actual timing of surgery in large centers is unclear. In this retrospective study, we aimed to determine the timing of surgery in a tertiary care spine center and the impact of early surgery on functional recovery in patients with MSCC.

## 2. Methods

### 2.1. Patient Population

**Figure 1:**
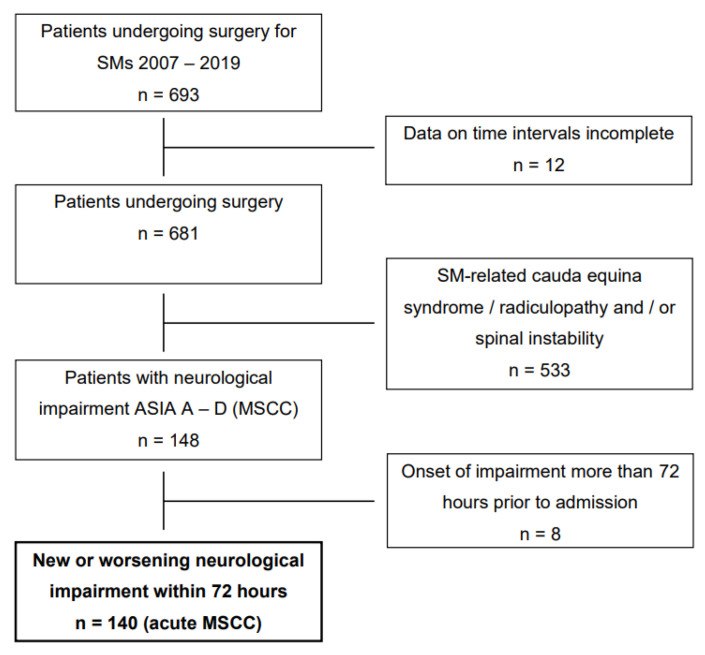
Flow chart illustrating patient groups. Bold type denotes subgroup with acute MSCC used for primary analyses. SM, spinal metastasis; MSCC, metastatic spinal cord compression.

We retrospectively reviewed data of all 693 patients admitted to undergo surgery for SMs at our institution between 2007 and 2019 (Figure 1). Of these, 681 patients had available documentation on symptom onset, timing of surgery, functional status on admission and discharge as well as imaging data. One hundred and forty-eight of these patients (21.7%) had symptomatic MSCC with neurological impairment as per the American Spinal Cord Injury Association (ASIA) grade D or lower; the remaining 533 patients (78.3%) had surgery for one or multiple different indications (such as SM-related spinal instability, cauda equina syndrome or radiculopathy, i.e., patients not fulfilling the aforementioned criteria), and were designated our non-MSCC study group. Epidural compression was assessed on preoperative magnetic resonance imaging (MRI), computed tomography (CT) or, in select cases, on post-myelography CT. In patients with multiple metastases located in different parts (cervical, thoracic, lumbar, sacral) of the spine, cases were assigned to the spinal part that was affected by the lesion most relevant to the surgical indication (Table 1). 

Most common primary entities in the acute MSCC group were prostate (25.0%), breast (15.7%), lung (12.1%), gastrointestinal tract (12.1%) and renal cell (7.6%) cancers. The proportions were similar for the non-MSCC group with prostate (20.5%), breast (19.0%), lung (11.1%), gastrointestinal tract (9.6%) and renal cell (7.6%) cancers, without significant difference between groups (*p* = 0.401). 

In 8 of the 148 patients with symptomatic MSCC, the symptom onset was more than 72 h prior to admission. In the remaining 140 patients (20.6%), a new neurological impairment corresponding to ASIA A–D or a deterioration of a pre-existing impairment to ASIA A-D had developed within 72 h prior to admission; these represented our primary study cohort (acute symptomatic MSCC; Figure 1). 

### 2.2. Surgical Treatment and Timing

As per standard of care, surgical decompression of the spinal cord was carried out at the symptomatic spinal levels affected by MSCC, i.e., usually at least one or multiple tumor laminectomies and, if needed, additional osteotomies (such as, e.g., pediculectomy or vertebral body replacement). In addition, pedicle screw instrumentation (typically percutaneously) was performed, usually including two spinal segments above and below the decompressed levels. Additional instrumentation of the anterior column (e.g., vertebral body replacement) was performed if indicated based on preoperative imaging, pre- and post-decompression instability, and the general condition and oncologic prognosis of the patient, either immediately or as a staged second surgery. The extent of the decompression, instrumentation and all surgical techniques complied with international guidelines and decision frameworks [12,13]. Due to the time-sensitive nature of surgical treatment, these judgements were frequently not made within the context of an interdisciplinary board meeting. Decisions on further adjunct treatment regimens after completion of surgery were, however, again according to standard of care. 

The exact times of admission to our hospital, surgical incision and discharge were drawn from the records. The time of symptom onset was established from the patient’s history and records of referring hospitals. The median time between admission to our hospital and surgical incision was analyzed post hoc and represented the basis for further stratification. For the primary outcome in the acute MSCC cohort, we compared the ordinal change in ASIA score by at least one grade between admission and discharge for two predefined subgroups: (1) the *early* subgroup undergoing surgery within the median time between admission and surgery as established for the entire acute MSCC cohort (2) the *late* subgroup undergoing surgery after the median. Further cutoffs were defined pre hoc as secondary outcomes—12 h and 24 h, respectively.

### 2.3. Statistical Analysis

For the primary outcome, we used chi-square testing to compare the proportions of improved ASIA grades between subgroups by discharge. Secondary analyses included comparison of occurrence rates of adverse events after surgery and Student’s t-test to compare metric items as well as Wilcoxon’s signed rank test to compare interval items between subgroups. We used IBM SPSS Statistics for Windows version 25.0 (Armonk, NY, USA 2017; https://www.ibm.com/products/spss-statistics (accessed on 1 February 2022)). The level of significance was defined a priori as *α* = 0.05. 

### 2.4. Ethical Considerations

All procedures were indicated and conducted in compliance with our department’s standards and the Declaration of Helsinki. The Ethics Committee Klinikum rechts der Isar of the Technical University Munich (Ethikkommission Klinikum rechts der Isar der Technischen Universität München) granted a positive vote (reference no. 96/19S) and waived the requirement for written informed consent.

## 3. Results

### 3.1. Patient Population and Timing of Surgery

We screened all 693 patients who had surgery for SM at our institution (cf. Methods; Figure 1). One hundred and forty (21.7%) of these had acute MSCC, defined as neurological impairment corresponding to ASIA grade D or lower acquired within 72 h before admission. All acute MSCC patients underwent surgery including decompression and, in the vast majority of cases (81%), pedicle screw instrumentation; 6% had additional vertebral body replacement. Surgery began within a median time of 16 h (interquartile range 10–22 h admission to surgical incision). This was the basis for defining the *early* (within 16 h) and *late* (after 16 h) surgery subgroups. Baseline characteristics of both the *non-MSCC* group and the *acute MSCC* group (*early* vs. *late* surgery) are shown in Table 1. Non-MSCC patients had surgery much later than acute MSCC patients (147 h; interquartile range 59–312 h; *p* < 0.001). As expected, the most common locations of the SM in acute MSCC were the thoracic (77.9%) and cervical (10.7%) spine, whereas there were more lumbar/sacral metastases in non-MSCC patients. The most common primary entities of both subgroups were prostate (early: 24.3% vs. late: 25.7%), breast (20.0% vs. 12.9%) and lung (12.9% vs. 11.4%) without statistically significant difference (*p* = 0.099). There were significantly more male patients in the acute MSCC group compared to the non-MSCC group. In the acute MSCC group, 33.6% of patients presented with ASIA grades A or B. The *early* and *late* acute MSCC subgroups did not differ with regards to age, sex, location of SM or initial ASIA grade (Table 1). There was no significant difference in the frequency of additional instrumentation between the *early* and *late* subgroups (*p* = 0.764). The *early* subgroup had a median hospital stay of 17 days compared to 16 days for the *late* subgroup (*p* = 0.527).

About half of the patients in the acute MSCC group were admitted within 24 h after symptom onset, the other half after 24–72 h (52% vs. 48%). In the entire cohort, the symptom onset was more than 28 days prior to admission in 31.4% (Table 2). When stratified by symptom onset to time of surgical incision, 16.4% of patients in the acute MSCC group underwent surgery within 24 h, 31.4% within 48 h and 52.1% within 72 h.

### 3.2. Correlation of Functional Recovery with Timing of Surgery

In acute MSCC, patients operated on before the median of 16 h after admission had a significantly higher rate of improvement of ASIA grades by discharge compared to those operated on later than 16 h after admission (Table 3).

The rates differed by a factor of about 2.5 (26.5% vs. 10.1% ASIA improvement). In the *early* subgroup, 16.2% improved by one ASIA grade and 10.3% by 2 grades, whereas in the *late* subgroup, 9.0% improved by 1 grade and only 1.1% by 2 grades. This benefit of early surgery increases when a cutoff of 12 h between admission and surgical incision is defined, for an improvement rate of 32.6% in the pre-12 h stratum compared to 11.0% after 12 h (*p* = 0.008; Table 3). 

With a cutoff of 24 h, no significant differences were found. A stratification by symptom duration also did not yield significant differences in the rates of ASIA grade changes (*p* = 0.271).

### 3.3. Correlation of Admission Time with Timing of Surgery

**Figure 2:**
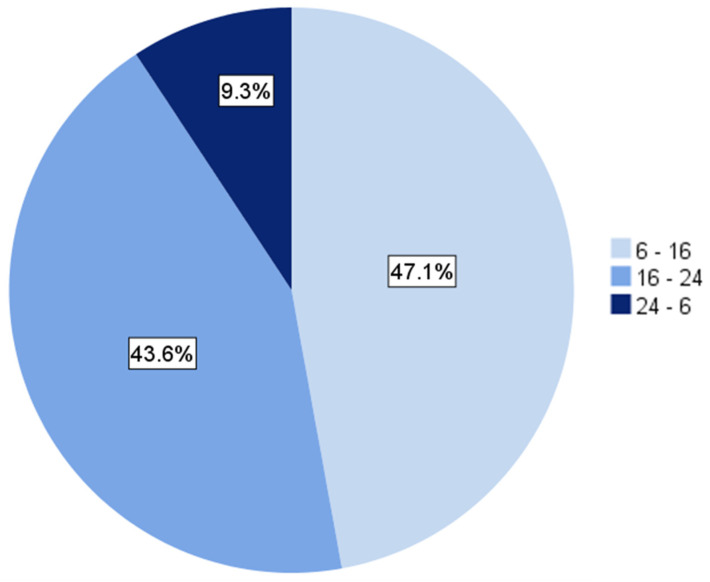
Proportions of patients in the acute MSCC subgroup admitted at different times of day.

When stratified by the time of day, most patients (47.1%) were admitted during regular working hours between 6 a.m. and 4 p.m. (Figure 2). The time of admission influenced the timing of surgery: patients admitted during the night shift, i.e., between 12 p.m. and 6 a.m., were significantly more likely to undergo *early* surgery (i.e., within the median; 92.3% vs. 7.7%; *p* < 0.001; Table 4).

### 3.4. Complications

Complication rates including mortality are reported in Table 5. When patients with acute MSCC were stratified by the timing of surgery, the *late* subgroup experienced a significantly higher rate of sepsis (7.4% vs. 2.2%; *p* = 0.027); other complication rates did not differ between the *early* and *late* surgery subgroups. The time of admission did not significantly affect any of the complication or mortality rates. Compared to patients undergoing surgery for spinal metastasis who did not suffer from acute MSCC, however, both the reoperation rate for surgical complications during the index hospital stay (14.4% vs. 7.6%; *p* = 0.014) and the ICU admission rate (12.2% vs. 6.6%; *p* = 0.032) were almost twice as high in the acute MSCC group (Table 5).

## 4. Discussion

### 4.1. A Case for Early Surgery in Patients with Acute MSCC 

This study was carried out to investigate the impact of surgical timing in acute MSCC. We found that the rate of recovery from severe neurological impairment depends on the time spent until surgery. In fact, the proportion of improved patients was increased by a factor of 2.6 in the group that received early surgery within the median 16 h (26.5% vs. 10.1%). While a cutoff of 12 h separated improved patients from unchanged or deteriorated patients even better (32.6% vs. 11.0%), a cutoff at 24 h did not.

This indicates that the time window permitting effective spinal cord decompression closes rapidly and it appears that “time is spinal cord” in these patients. This strongly argues for a treatment strategy aiming at the earliest possible surgery in acute MSCC 24 h, seven days a week, and the goal would always be to initiate surgery within 24 h.

In our department, it has been standard practice to aim for early surgery including decompression and instrumentation in acute MSCC. This means that patients are operated on hours after admission at the latest. Patients getting ready for surgery during regular operating hours would usually be operated on the same day after regular surgeries are finished, and those arriving later during the day or at night would either be operated on during the night or during the regular operating hours of the next day, depending on anesthesia capacities during the night. This certainly leaves room for even earlier surgery in many cases. 

One might assume that such an “ultra-early surgery” treatment paradigm could entail side effects negatively affecting the patient, such as an increase in adverse events. However, in this study, complications and especially revision surgeries did not occur more frequently in the *early* subgroup. The fundamental paradigms underlying surgical treatment of SMs are symptom control, spinal stabilization and preservation of neurological function [12,14,15,16,17,18]. The guidelines available to treating specialists generally regard surgery as a first step in the oncological treatment regimen, laying the foundation for adjunct radiation and systemic treatment tailored to the burden of disease and the primary tumor entity, optimally through a network of oncological specialists [12,13,19]. Maintaining functional autonomy is crucial for patients; not only does it secure quality of life, but often it enables the feasibility of said adjunct treatment regimens in the first place [15,20,21,22,23]. It follows that patients with acute neurological impairment due to metastatic spinal cord compression are particularly prone to remain or to become unfit for further treatment and, given our findings, that early surgery may be even more important in these cases. 

### 4.2. Instrumentation for Spinal Stabilization in Patients with Acute MSCC

In our department, posterior pedicle screw instrumentation in addition to decompression is the standard procedure in acute MSCC. This treatment paradigm is based on several aspects, several of which have emerged recently. 

First, there have always been cases that require immediate spinal stabilization, e.g., when tumor-related instability has led to spinal deformity causing spinal cord compression. 

Secondly, as mentioned above, the life expectancy of patients with SMs has increased. This means that tumor-related as well as decompression-related instability that might not be relevant in the short term need to be addressed in order to ensure mid- and even long-term pain relief and functional independence. Spinal cord decompression via tumor laminectomy renders the affected segments of the spine less stable, promoting sagittal malalignment such as post-laminectomy kyphosis that is associated not only with pain, but also with neurological deterioration. This particularly holds true for junctional segments, where often not only posterior, but also anterior column stabilization may be warranted [24,25]. 

Thirdly, the advent of minimally invasive approaches including the routine use of navigational systems and specialized pedicle screw systems have drastically facilitated spinal instrumentation and improved procedure-related safety and efficacy [2,26,27]. These developments have rendered posterior pedicle screw instrumentation in addition to decompression the standard of care in many centers across a wide range of SM patients [3,12,19,28,29]. 

Naturally, the decision for additional instrumentation must be based on weighing these substantial benefits against potential disadvantages associated with more extensive surgery. The patient’s individual performance status, general condition and burden of the systemic disease must always be taken into account [8,12,18,30]. A staged approach after initial pedicle screw instrumentation and decompression of MSCC may for instance comprise the initiation of adjunct radiation therapy before conducting delayed anterior instrumentation or addressing asymptomatic but nonetheless unstable lesions [7,15,28]. In our department, we have refrained from immediate instrumentation in cases that are unlikely to become unstable even with tumor laminectomies (e.g., in patients with osteoblastic metastases, or with sufficient fusion already present). In patients with extremely poor general condition requiring the least invasive surgery possible, we typically have aimed at staged instrumentation when tumor-related or decompression-related instability was present. 

At any rate, it is crucial to adopt a standard of treatment guideline for clinical decision-making in such scenarios. Surgical treatment should be integrated into an overarching interdisciplinary oncological treatment regimen whenever possible, as one of the primary goals is to preserve or restore functional competence of patients in order to be able to undergo radiation and systemic therapies [8,9,12,19,31]. Several guidelines are available to aid in these decision-making processes, such as the NOMS framework [14]. Of note, because of the lack of reliable data, these eschew providing clear recommendations on the timing of surgical intervention.

### 4.3. Comparison with Previous Studies and Outlook

In contrast to traumatic spinal cord injury, which has repeatedly been demonstrated to require timely surgical intervention [11,32,33], there is little evidence concerning the timing of surgery in acute MSCC, and what is available originates from retrospective investigations [1,34,35,36]. This is mirrored in vague recommendations in current practice guidelines that eschew any explicit recommendation regarding the timing of surgical intervention [37]. More recently, prospective cohort studies were able to demonstrate superior neurological outcomes including the ability to ambulate with early surgery, although the evidence level remains low due to very limited cohort sizes [35,38]. Van Tol et al. were able to analyze a sizable series and found that timely surgery may lead to better quality of life and higher survival rates; their study, however, is limited by tremendous variation in the allocation to their delayed and early arms based on a loose definition at the discretion of the attending surgeon [17]. 

Even though the present study is also based on a monocentric retrospective analysis, which is obviously its most significant limitation, we are confident that our findings add to the evidence supporting early surgery in acute MSCC given that we report the largest series to date and that we are able to delineate, for the first time, a reasonable time cutoff (the impact of surgery was even more pronounced with 12 h than with the median 16 h after admission, and there was no difference with a cutoff of 24 h after admission any more). This will provide the framework for a much-needed prospective study that may eventually inform practice guidelines and lead to implications for both in-house treatment strategies as well as patient referral strategies within the hospital network aiming at early recognition and transfer of patients in need of rapid decompressive surgery. The potential benefit may represent the difference between a patient being able to walk on their own and a patient being bedridden for the rest of their life. This in itself harbors a multitude of implications for the patients’ ability to pursue activities of daily life, their functional independence, health-related quality of life and fitness to undergo adjunct systemic oncological treatment and, eventually, patient survival [17,39].

## 5. Study Limitations

By nature, retrospective analyses may be limited by imprecise data, particularly concerning time periods. This likely explains why we were not able to find a significant impact of symptom duration, which is, at the resolution of hours, much more difficult to narrow down than the time between admission and surgery in a retrospective series. Consequently, prospective studies are needed to confirm our findings. Until then, the significant outcome difference between the early and late surgery groups in the present study warrants a treatment strategy aiming at surgery as fast as feasible and possible in acute MSCC, without jeopardizing the patients’ safety. 

## 6. Conclusions

In this largest cohort study of patients with acute MSCC to date, early surgery within 16 h or even 12 h appears to substantially increase the chance of functional recovery without increasing the risk of serious peri- and postoperative complications. Prospective studies are needed to establish a distinct cutoff and high-quality evidence regarding the impact of early surgery on both neurological outcome as well as adverse events. Until then, surgical treatment strategies should aim at early intervention in these patients given consistent findings in retrospective series. 

## Figures and Tables

**Table 1:** Baseline characteristics of both the non-MSCC as well as the acute MSCC groups. Early and late subgroups within acute MSCC defined by surgery within or after the median time from admission to surgery; N—number; *p*—level of significance; bold typeface denotes statistically significant difference.

Variation		Acute MSCC		*p* *Early/Late*	Non-MSCC *n* = 533	*p* *MSCC/Others*
		Early *n* = 70	Late *n* = 70			
**Mean age in years** **(range)**		65.2 (24.3–94.3)	69.1 (15.9–93.6)	0.637	65.7 (15–94)	0.255
**Sex** **(n; % of subgroup)**	Male	49	50	0.702	315	**0.007**
		(70.0%)	(71.4%)		(59.1)	
**Localization** **(n; % of subgroup)**	Cervical	6	9	0.476	40	**<0.001**
		(8.6%)	(12.9%)		(7.4)	
	Thoracic	57	52		204	
		(81.4%)	(74.3%)		(37.7)	
	Lumbar	0	2		163	
		(0%)	(2.9%)		(30.1)	
	Cervicothoracic	3	3		51	
		(4.3%)	(4.3%)		(9.4)	
	Thoracolumbar	4	4		41	
		(5.7%)	(5.7%)		(7.6)	
	Lumbosacral	0	0		42	
		(0)	(0)		(7.7)	
**Mean hours admission–cut (hours)**		9.6	27.8	**<0.001**	245.7	**<0.001**
**ASIA on admission (*n*; % of subgroup)**	A	10	12	0.752	-	-
		(14.3%)	(17.1%)			
	B	14	11			
		(20.0%)	(15.7%)			
	C	16	18			
		(22.9%)	(25.7%)			
	D	30	29			
		(42.9%)	(41.4%)			
	E	0	0			
		(0.0%)	(0.0%)			

**Table 2:** Times of symptom onset prior to admission for acute MSCC subgroup and entire cohort.

Symptom Onset	Acute MSCC		All	
	*n*	%	*n*	%
Unknown			38	5.6
<6 h	23	16.4	32	4.7
6–24 h	44	31.4	64	9.4
24 h–3 d	73	52.2	95	14.0
3 d–7 d			57	8.4
7 d–28 d			181	26.6
>28 d			214	31.4

**Table 3:** Changes in ASIA grades by discharge in patients with acute MSCC, stratified by different surgical timing cutoffs: left column, by median time interval from admission to surgery (i.e., within vs. after 16 h); middle column, within vs. after 12 h; right column, within vs. after 24 h. Bold typeface denotes statistically significant difference.

Variation		Early *n* = 70	Late *n* = 70	*p*	<12 h *n* = 46	>12 h *n* = 94	*p*	<24 h *n* = 125	>24 h *n* = 15	*p*
**ASIA** **change by discharge**	Same (%)	70.6	81.2	**0.026**	63.0	83.0	**0.006**	77.6	66.7	0.617
	Worse (%)	2.9	8.7		4.3	6.4		5.6	6.7	
	Better (%)	26.5	10.1		32.6	10.6		16.8	26.7	
**ASIA** **grades by discharge**	A (%)	13.8	21.4	**0.010**	23.9	8.5	**0.011**	12.8	6.7	0.491
	B (%)	15.7	9.6		8.7	10.6		11.2	0.0	
	C (%)	20.0	31.4		8.7	6.4		8.0	13.3	
	D (%)	37.1	34.3		10.9	31.9		25.6	20.0	
	E (%)	14.3	4.3		47.8	42.6		42.4	60.0	

**Table 4:** Patients with acute MSCC: Timing of surgery in relation to time of admission to hospital. Bold typeface denotes statistically significant difference.

Variation		Time of Admission (Hours)						*p*
		6–16		16–24		24–6		
**Timing of surgery**	**Early** (*n*; % of column)	26	(39.4%)	32	(52.5%)	12	(92.3%)	**<0.001**
	**Late** (*n*; % of column)	40	(60.6%)	29	(47.5%)	1	(7.7%)	

**Table 5:** Complication rates after surgery, stratified by timing of surgery, time of admission and for acute MSCC versus non-MSCC patients. ICU—intensive care unit; bold typeface denotes statistically significant difference.

Variation	Timing of Surgery			Time of Admission				MSCC vs. Others		
	Early	Late	*p*	6:00–16:00	16:00–24:00	24:00–6:00	*p*	Acute MSCC	Non-MSCC	*p*
**Reoperation (** **%)**	13.0	15.7	0.654	16.7	13.3	7.7	0.668	14.4	7.6	**0.014**
**Pneumonia (** **%)**	10.9	7.4	0.511	13.0	5.8	8.3	0.441	9.3	5.3	0.095
**Thromboembolic event (** **%)**	1.6	1.9	0.903	3.7	-	-	0.300	1.7	5.5	0.084
**Sepsis (** **%)**	2.2	7.4	**0.027**	1.9	5.8	-	0.425	3.4	1.0	0.053
**ICU stay (** **%)**	12.9	11.5	0.810	8.2	17.5	7.7	0.262	12.2	6.6	**0.032**
**Mortality (** **%)**	3.0	6.6	0.340	4.9	5.6	-	0.692	4.7	2.7	0.251

## Data Availability

Data is available in the text.
